# Examining relationship between occupational acid exposure and oral health in workplace

**DOI:** 10.1186/s12889-020-09496-6

**Published:** 2020-09-07

**Authors:** Wei-Liang Chen, Yuan-Yuei Chen, Wei-Te Wu, Ching-Huang Lai, Yu-Shan Sun, Chung-Ching Wang

**Affiliations:** 1 Division of Family Medicine, Department of Family and Community Medicine, Tri-Service General Hospital, and School of Medicine, National Defense Medical Center, Taipei, Taiwan, Republic of China; 2 Division of Occupational Medicine, Department of Family & Community Medicine, Tri-Service General Hospital, National Defense Medical Center, Taipei, Taiwan, Republic of China; 3 Department of Pathology, Tri-Service General Hospital; and School of Medicine, National Defense Medical Center, Taipei, Taiwan, Republic of China; 4 Department of Pathology, Tri-Service General Hospital Songshan Branch; and School of Medicine, National Defense Medical Center, Taipei, Taiwan, Republic of China; 5 National Institute of Environmental Health Sciences, National Health Research Institutes, Miaoli, Taiwan, Republic of China; 6 School of Public Health, National Defense Medical Center, Taipei, Taiwan, Republic of China

**Keywords:** Acid mist, Tooth erosion, CPITN, Loss of attachment

## Abstract

**Background:**

Acid mist can suspend in the air and enter the body via skin contact, the respiratory tract, or even oral intake, which pose various health hazards. Previous studies have shown that occupational exposure to acid mist or acidic solutions is a major risk factor for oral diseases. However, the findings are inconsistent and do not consider individual factors and lifestyles that may cause the same oral diseases. Therefore, we conducted a comprehensive oral health survey and collected detail information to confirm the effect of acidic solution exposure on worker’s oral health.

**Methods:**

From 4 acidic solution factories, a total of 309 subjects (157 in control and 152 in exposed group) was enrolled. All participants competed oral examinations and self-report questionnaire, including the decayed, missing, and filled teeth (DMFT) index, community periodontal index (CPI), loss of attachment (LA) index, and tooth erosion. Multivariate logistic regression analysis was used to determine the association between the acidic solution exposure and oral health.

**Results:**

The results showed that acid exposure was correlated with soft oral tissue injury rather than hard oral tissue in our survey. In the multivariate model (adjusted for sex, age, worked years, education level, mouthwash use, dental floss use, tooth brushing, mask use, smoking, drinking, chewing areca and dietary habits with acidic foods), significant relationships of acid exposure with LA score were observed (OR = 2.32, 95% CI 1.03–5.26). However, the presence of acid exposure was not significantly associated with tooth erosion, DMFT, and CPITN.

**Conclusion:**

Our study highlighted that occupational acid exposure was an independent risk factor for periodontal health, especially LA. It is important to strengthen occupational hazard control, educate workers on oral disease and related factors, and raise the awareness of oral hygiene.

## Background

Acidic solution work generally refers to any work where workers may be exposed to acidic substances or related derivatives. Acidic solutions may be present in the air in three different forms: mist, vapor, and gas. Industries exposed to acidic solutions include manufacturing (phosphate fertilizers, isopropyl alcohol, ethanol, sulfuric acid, nitric acid, and lead-acid batteries), construction, petroleum and coal products, oil and gas extraction, printing and publishing, paper-making, and leather manufacturing. In addition, workers who engage in the industries involved in metal materials or metal-related compounds, such as smelting copper, electroplating, pickling and other metal surface treatment industries, are more likely to be exposed to metal-containing acid mist. Acid mist can suspend in the air and enter the body via skin contact, the respiratory tract, or even oral intake, which pose various health hazards, such as respiratory irritation [1], oral lesions, periodontal disease [2], tooth erosion [3], and may even increase the risk of cancer [4–6]. Dental illnesses, although common, are important health problems that tend to be ignored.

In addition to affecting chewing and eating, dental diseases may cause or worsen chronic diseases over the long term [7, 8]. For example, periodontal disease may increase the risk of cardiovascular [9] and neurodegenerative diseases [10], affect glycemic control [11], and increase the risk of infants with a low birth weight [12]. Previous studies have shown that occupational exposure to acid mist or acidic solutions is a major risk factor for oral diseases [13–15]. However, the findings are inconsistent and do not consider individual factors and lifestyles that may cause the same oral diseases. The aim of the study was to investigate the relationship between occupational acidic solution exposure and oral health in a Taiwanese adult population.

## Methods

### Study population

Based on the directory of factories and manufacturers from the Ministry of Economic Affairs of the Republic of China, we selected enterprises in the metal surface treatment or electroplating industries in municipalities and counties in Taiwan that employed 300 or more employees and were willing to participate in this survey. In this study, we selected 4 acidic solution factories willing to collaborate since July to November, 2016. The factories were in Northern Taiwan, and the workers may have been exposed to different acidic solutions, including hydrochloric acid, sulfuric acid, and nitric acid. Annual reports of work environments were summarized in Table 1. The total numbers of acid non-exposed and exposed workers joined the study were estimated to be 5100 and 500.

**Table 1:** Acid mist assessment of the study factory

	Factory A		Factory B	
	Air sampling (mg/m^3^)	PEL-TWA^#^ (mg/m^3^)	Air sampling (mg/m^3^)	PEL-TWA (mg/m^3^)
Nitric acid	0.014	5.2	–	–
Sulfuric acid	< 0.007	1	0.066	1
Hydrochloric acid	< 0.050	7.5	–	–

#*PEL* Permissible exposure limit-time weighted average

#One study factory did not provide acid mist assessment papers; another study factory did not enroll exposed employees

**Fig. 1:**
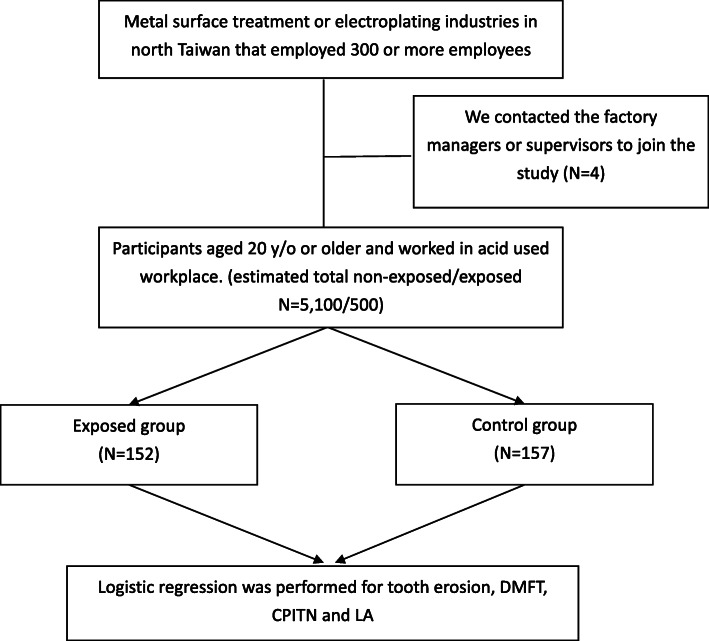
Flow chart which represented the steps of analysis performed in the study

We contacted the factory managers or supervisors, who in turn contacted and confirmed which study subjects were willing to participate. We then conducted the survey at the time of a routine health check at each factory. Study subjects were workers aged 20 years or older who were enrolled at a 1:1 ratio into exposure and control groups that were defined by work history information. The exposed groups comprised workers engaged in acid mist work, while controls were subjects conducting similar tasks but who were not exposed to acids (e.g., other production line workers). All participants completed oral examinations and self-report questionnaire. A total of 309 subjects (157 in control 21 and 152 in exposed group) participated was enrolled in the further analysis (Fig. 1). The study was reviewed and approved by the Institutional Review Board (IRB) of the Tri-Service General Hospital (TSGHIRB No.: 1–105–05-080) before the study began. Each subject signed the informed consent form at the time of enrollment.

### Oral examination

Each enrolled subject underwent a comprehensive examination of their teeth and oral mucosa by a team of qualified and uniformed trained dentists to minimize the variation caused by different dentists. The detailed results were recorded in the subject’s medical records for subsequent data analysis. To reduce error, the dentists were unaware of each subject’s occupational exposure. A mouth mirror and a probe were used to record the decayed, missing, and filled teeth (DMFT) index based on WHO criteria [16]. The community periodontal index (CPI) and loss of attachment (LA) index were measured for assessment of periodontal status, by using a mouth mirror and a specifically designed periodontal probe [17]. The measured variables included tooth erosion (Keels-Coffield clinical severity scale [18]) (Supplemental table 1), tooth abrasion, tooth decay (DMFT), and periodontal disease (CPI and LA).

The DMFT corresponds to the average number of decayed, missing, and filled permanent teeth. The WHO CPI [19] and LA [20] levels were used to assess periodontitis (Hu-Friedy PCP11.5B CC SE probe 3.5–5.5-8.5-11.5). Periodontitis was defined as a CPI or LA value greater than or equal to 1 (Supplement table 2). The index teeth numbers were 11, 16, 17, 26, 27, 31, 36, 37, 46 and 47. A CPI was used at the gingival margin as a reference to measure the periodontal pocket. During the measurement, the probe was aligned with the long axis of the tooth and the total extent of the pocket was explored. The results were recorded as follows: 0: the 1st marking of probe remained completely visible, without bleeding after probing - healthy gum; 1: the 1st marking of probe remained completely visible, with bleeding after probing - gingivitis; 2: the 1st marking of probe remained completely visible, with dental calculus (including supra-or subgingival calculus); 3: the 2nd marking of probe remained completely visible - shallow pocket; and 4: the 3rd marking of probe remained completely visible - deep pocket. An LA was used at the cemento-enamel junction (CEJ) as a reference to measure the periodontal pocket. During the measurement, the probe was aligned with the long axis of the tooth and the total extent of the pocket was explored. The results were recorded as follows: 0: CEJ was within the first marking; 1: CEJ reached the second marking; 2: CEJ reached the third marking; 3: CEJ reached the fourth marking; and 4: CEJ reached beyond all markings (Supplemental table 1).

### Covariables assessment

Demographic characteristics in the present study were obtained from participants by self-structured questionnaire (supplemental questionnaire). Educational level was divided into studying less than 12 years and more than 12 years. Acidic foods were defined as foods where the pH value was ≤6 [21]. For dietary habits with acid foods, a type of acidic food was only considered when the subject consumed that acidic food more than once per week. Owing to the absence of data regarding dietary habits with acidic foods, we pooled each type of acidic foods and categorized into four groups (Q1 to Q4). We collected information such as cigarette smoking, alcoholic drinking, chewing areca, teeth brushing, mouthwash use, dental floss use, mouth breathing, and halitosis from self-reported questionnaire that participants answered yes or no.

### Statistical analysis

IBM SPSS Statistic version 20 was used for statistical analysis. For descriptive analyses, the t-test, one-way analysis of variance, and chi-square test were performed for comparative analyses of the demographics. Moreover, the frequency, and the severity of tooth erosion, DMFT, CPI and LA scores were analyzed. Both t-tests and chi-square tests were performed to investigate the relationship between demographic characteristics and oral health. Multivariable adjustment was done by applying extended model. Model 1: adjusted for sex, age, worked years and education level; Model 2: adjusted for model 1+ mouthwash use, dental floss use, tooth brushing, mask use, smoking, drinking, and chewing areca. Model 3: adjusted for model 2+ dietary habits with acidic foods. Moreover, multivariate logistic regression analysis was used to investigate the association of occupational acid exposure with tooth erosion, DMFT, CPI, and LA. A significance level of < 0.05 was adopted for this study.

## Results

The basic demographics are shown in Table 2. Demographic characteristics in the present study were obtained from participants by questionnaire, including age, sex, educational level, work years, personal illness history, cigarette smoking, alcoholic drinking, chewing areca, dental habits, and dietary habits with acidic foods. In the control group, 26.8% of the subjects were women and 73.2% were men. In the exposure group, 17.1% were female and 82.9% were male. The control group was aged 37.76 ± 10.06, and the exposure group was aged 30.08 ± 6.43. The subjects in the control and exposure groups had worked 10.41 ± 10.80 and 2.24 ± 2.42 years, respectively. In the control group, the body mass index (BMI) values were 24.47 ± 4.16, and the BMI values in the exposure group were 23.70 ± 3.58. In the control group, 34.5% of the subjects completed secondary education or below (≤ 12 years) and 65.5% completed tertiary education or above (> 12 years). The 47.2 and 52.8% of the exposure group subjects completed secondary and tertiary education, respectively. These differences were statistically significant. In the personal health behavior, only chewing areca had significant higher prevalence in the control group. In the personal oral health habits, mask use, teeth brushing, and mouthwash use were significant higher in acid mists exposed group. Inversely, dental floss use was significant higher in control group. The total number of types of acidic food was also showed significant difference between control and exposed group.

**Table 2:** Distribution of demography characteristic in the study population between exposed and control group

	Variable	Control group *N* = 157	Exposed group *N* = 152	*p*-value
Sex				0.04*
	Female	26.8%	17.1%	
	Male	73.2%	82.9%	
Age		37.76 ± 10.06	30.08 ± 6.43	< 0.01*
Worked years		10.41 ± 10.80	2.24 ± 2.42	< 0.00*
#BMI		24.47 ± 4.16	23.70 ± 3.58	0.09
Education				0.03*
	≤ 12 years	34.5%	47.2%	
	> 12 years	65.5%	52.8%	
Smoking				0.92
	No	60.3%	60.8%	
	Yes	39.7%	39.2%	
Drinking				0.08
	No	65.2%	55.2%	
	Yes	34.8%	44.8%	
Chewing areca				0.01*
	No	87.1%	95.8%	
	Yes	12.9%	4.2%	
Mask use				< 0.00*
	No	26.5%	4.2%	
	Yes	73.5%	95.8%	
Teeth brushing				< 0.00*
	Less than twice a day	88.5%	55.0%	
	Twice a day or more	11.5%	45.0%	
Mouthwash use				< 0.01*
	No	78.8%	62.4%	
	Yes	21.2%	37.6%	
Dental floss use				< 0.00*
	No	34.8%	87.2%	
	Yes	65.2%	12.8%	
Mouth breathing				0.12
	No	61.5%	52.7%	
	Yes	38.5%	47.3%	
Halitosis				0.10
	No	50.0%	59.5%	
	Yes	50.0%	40.5%	
##Dietary habits with acidic foods (Q1 VS Q4)				< 0.000*
	Q1	24.8%	31.1%	
	Q2	36.3%	17.4%	
	Q3	22.9%	15.9%	
	Q4	15.9%	35.6%	

*p* < 0.05 by t-test or chi-square test between exposed and control groups

*p* < 0.05 by one-way analysis of variance (ANOVA) for dietary habits with acidic foods

#*BMI* Body mass index

## The total number of types of acidic food which subjects consumed more than once per week. We separated the data into 4 Quarter (Q1 to Q4) for analysis

*p < 0.05

In the Table 3, the distribution of oral hard/soft tissue indices between acid exposed and control group was showed. In the control group, the workers had higher prevalence of oral hard tissue problems compared with acid exposed group, especially noted in DMFT indices (*p* <  0.01). Inversely, the workers had lower prevalence of oral soft tissue problems (*p* = 0.02 in CPITN indices and p <  0.01 in LA indices) compared with acid exposed group.

**Table 3:** Distribution of oral hard/soft tissue indices between acid exposed and control group

Group	Control group N = 157		Exposed group N = 152		***P*** Value
	No	Yes	No	Yes	
**Oral hard tissue**					
**Tooth erosion**	Level 0	Level 1 to 3	Level 0	Level 1 to 3	
	66.7%	33.3%	74.8%	25.2%	0.12
**DMFT**	DMFT = 0	DMFT> 0	DMFT = 0	DMFT> 0	
	5.7%	94.3%	15.8%	84.2%	< 0.01**
**Oral soft tissue**					
**CPITN**	CPITN = 0	CPITN> 0	CPITN = 0	CPITN> 0	
	12.4%	87.6%	4.6%	95.4%	0.02*
**LA**	LA = 0	LA > 0	LA = 0	LA > 0	
	59.5%	40.5%	42.4%	57.6%	< 0.01**

#*p* < 0.05 by chi-square test between exposed and control groups

*p < 0.05, **p < 0.01

Abbreviation: *DMFT* decayed, missing, and filled teeth; *CPITN* community periodontal index of treatment needs; *LA* loss of attachment

The unadjusted and adjusted odd ratios (OR) of the acid exposed status and demography characteristic on tooth erosion, DMFT indices, CPITN and LA were showed in Table 4. In the unadjusted analysis, the result found that acid exposed workers had positive association with CPITN (2.92, 95%CI 1.19–7.16) and LA score (1.20, 95% CI 1.26–3.15). However, acid exposed worker had negative association with DMFT score (0.32, 95% CI 0.15–0.72). In the adjusted analysis, tooth erosion, DMFT and CPITN had no significant difference was observed after adjusted for sex, age, worked years and education level (model 1). However, significant relationships of acid exposure with LA score were observed in model 1 (OR = 3.18, 95% CI 1.65–6.15), model 2 (OR = 2.28, 95% CI 1.01–5.11), and model 3 (OR = 2.32, 95% CI 1.03–5.26). Collectively, the presence of acid exposure was significantly associated with LA, but not tooth erosion, DMFT, and CPITN after the adjustment of pertinent covariates.

**Table 4:** Association among the acid exposed status and tooth erosion, DMFT indices, CPITN and LA

	Unadjusted Model OR (95% CI)	***P*** Value	Model 1 OR (95% CI)	***P*** Value	Model 2 OR (95% CI)	***P*** Value	Model 3 OR (95% CI)	***P*** Value
	**Tooth erosion**							
Acid exposure	0.67 (0.41–1.11)	0.12	0.70 (0.36–1.40)	0.30	0.54 (0.23–1.24)	0.15	0.49 (0.21–1.15)	0.10
	**DMFT**							
Acid exposure	0.32 (0.15–0.72)	0.01*	0.82 (0.30–2.22)	0.70	0.91 (0.27–3.11)	0.88	0.89 (0.26–3.06)	0.86
	**CPITN**							
Acid exposure	2.92 (1.19–7.16)	0.02*	2.33 (0.81–6.69)	0.12	0.99 (0.26–3.79)	0.98	1.05 (0.27–4.03)	0.94
	**LA**							
Acid exposure	1.20 (1.26–3.15)	< 0.01**	3.18 (1.65–6.15)	< 0.01**	2.28 (1.01–5.11)	0.05*	2.32 (1.03–5.26)	0.04*

#Model 1: adjusted for sex, age, worked years and education level

#Model 2: model 1+ mouthwash use, dental floss use, tooth brushing, mask use, smoking, drinking, and chewing areca

#Model 3: model 2+ dietary habits with acidic foods

#Univariate and multivariate logistic regression model were used

*p < 0.05, **p < 0.01

Abbreviation: *DMFT* decayed, missing, and filled teeth; *CPITN* community periodontal index of treatment needs; *LA* loss of attachment

## Discussion

Results from our study showed that strong inorganic acids, such as hydrochloric acid, nitric acid, or sulfuric acid, used in industrial processes have a positive association with LA. Emerging evidence point out LA is a kind of severe periodontal disease and a potential effect of exposure to acid mists [22]. This result was similar with the findings of Finnish and Brazil study. The results from the Finnish study showed that among workers with occupational exposure to sulfuric acid, 36.9% developed periodontal pockets (periodontal pockets detected by LA); the ratio was merely 30.9% in the control group [22]. In Brazil study, periodontal attachment loss was observed in workers who had acid mists exposure and did not use dental floss [23]. Recent studies also showed that acidic solutions in the work environment increase the risk of oral soft tissue (oral mucosa and periodontal tissue) diseases, such as mucosal ulcers [13], which can increase the risk of periodontal disease [23], oral fibrosis and stomatitis, leading to periodontal pocket induction and loss of attachment. Although typical periodontal disease should have gingival bleeding and periodontal pockets, another indicator of periodontal disease, CPITN, can connect these two aspects. A previous study had used CPITN as an indicator for acid mists related periodontal change [2]. The results were only showed significant difference in unadjusted model but not statistically differences after considering numerous covariates in the analysis. The reason could be explained by good oral hygiene and mask use in our acid exposed group.

We infer that acidic solutions can cause oral soft tissue lesions by one or more of the following mechanisms: 1) acidic solutions directly irritate soft oral tissue, such as the gums and periodontal tissue, and can directly disrupt cell function and arrangement of the soft tissue [2]; 2) acidic solutions may suppress the immune-protective components of saliva, thus indirectly affecting gingival or periodontal immunity, resulting in persistent gingival or periodontal inflammation, and aggravating periodontal disease; and 3) acidic solutions may damage the ability of saliva to balance the pH, resulting in an acidic oral environment, which, together with poor personal oral hygiene, smoking, drinking, and chewing areca, may cause bacteria to grow, thereby damaging the gums or periodontal tissue [24]. On the other hand, some researchers investigated periodontal tissue health and found that occupational acid exposure was not significantly related to periodontal disease [2, 25]. As a result, more long-term follow-up studies are needed to clarify the relationship between different occupational acid exposures and oral soft tissue damage.

Previous studies showed that besides periodontal disease, occupational exposure to acid mist and acid solution may cause tooth damage [3, 23, 25–30], especially tooth erosion [2]. In 2010, a Japanese study showed that the mean prevalence rate of tooth erosion among battery factory workers was 22.5%, which was also proportional to work history. In a report by Chikte et al. [31], clinical examination showed that among electroplating workers, 60% had toothache and sensitivity, 76% had varying degrees of loss of tooth structure, and 25% had occupational tooth loss [31]. Petersen et al. [30] reported that due to exposure to sulfuric acid mist, 56% of battery factory workers had tooth thinning and tapering, 29% reported tooth shortening, and 31% had tooth erosion; however, this study did not confirm the effect of occupational acid exposure on tooth erosion. In 1984, Gamble et al. [32] showed a strong correlation between exposure to sulfuric acid mist and tooth erosion; tooth damage occurred as early as 4 months after a mean sulfuric acid exposure of 0.23 mg/m^3^. In this study, the mean sulfuric acid exposure in the factories included in the survey was less than 0.066 mg/m^3^, which may be related to more advanced engineering control of occupational acid mist exposure, the awareness of oral hygiene, and the use of protective equipment, resulting in a great reduction in occupational hazards. As a result, this study showed no significant correlation between acid exposure and tooth erosion. In the evaluation of acidic exposure related dental caries, we used the mean DMFT score as a marker. However, dental caries was not correlated with acidic exposure in our study population. The same results were also found in the UK [25], Japan [33] and Brazil [3] caries experience studies in acid workers.

Another interesting point in our study was the use of masks did not significantly reduce the risks. Personal protective equipment is a worker’s last line of defense against workplace hazards, especially when all other controls set up to minimize risk and protect the worker have been exhausted. However, the efficiency of personal protective equipment is significantly reduced if it is worn incorrectly or if it does an inadequate selection. Employees need to be adequately educated the potential hazards and trained in how to wear protective equipment in workplace.

The limitation of our study included a cross-sectional study design and reporting bias of self-reported questionnaire. Diabetes would be an important factor for CPI. However, there were only 5 diabetes patients in our study population (data do not show). The reason might be due to related young adult. Therefore, history of diabetes was not considered as a confounder for CPI. In the present study, we recruited voluntary participants instead of randomized sample population. Selection bias might be occurred due to the recruitment strategy in the present study. In addition, socioeconomic status, which might be the potential confounding factor, was unavailable from the survey. We adjust educational level, which is associated with economic and social outcomes [34], for the association between acid mist exposure and dental illness.

## Conclusion

This study showed that occupational acid exposure during acidic solution work was significantly associated with periodontal health, especially LA. It is important to strengthen occupational hazard control, educate workers on oral disease and related factors, and raise the awareness of oral hygiene, which are all the best solutions to improve the oral health of these workers.

## Supplementary information

**Additional file 1: Table S1.** Diagnostic criteria of oral health in the study

**Additional file 2: Table S2.** The oral hard/soft tissue indices for study survey

**Additional file 3.** Questionnaire. English version of personnel health survey questionnaire

## Data Availability

Yes, all data are fully available without restriction. Please contact with corresponding author.

## Abbreviations

DMFT: Decayed, missing, and filled teeth
CPITN: Community periodontal index of treatment needs
LA: Loss of attachment
CEJ: Cemento-enamel junction
