# Supplementary material for: Examining relationship between occupational acid exposure and oral health in workplace
Source: BMC Public Health. 2020 Sep 7;20:1371. doi: 10.1186/s12889-020-09496-6 (PMC7487460; doi:10.1186/s12889-020-09496-6)
Supplement: Supplementary file 1 — Additional file 1: Table S1. Diagnostic criteria of oral health in the study [file 12889_2020_9496_MOESM1_ESM.doc]

**Supplemental table 1. Diagnostic criteria of oral health in the study**

**Criteria of the Keels-Coffield clinical severity scales for dental erosion**

| Level | Description |
| --- | --- |
| 0 | No erosion |
| 1 | Mild: Only the cusp tips are affected; shallow moon craters are present. |
| 2 | Moderate: Deep moon craters or depressions are preset and may coalesce. |
| 3 | Severe: Teeth are slick with little or no anatomy present; possible pulpal exposures. |

**Criteria of CPITN score for periodontal disease**

| Score | Description |
| --- | --- |
| 0 | The 1st marking of probe remained completely visible, without bleeding after probing |
| 1 | The 1st marking of probe remained completely visible, with bleeding after probing |
| 2 | The 1st marking of probe remained completely visible, with dental calculus (including supra-or subgingival calculus) |
| 3 | The 2nd marking of probe remained completely visible |
| 4 | The 3rd marking of probe remained completely visible |

**Criteria of LA score for loss of attachment in periodontal disease**

| Score | Description |
| --- | --- |
| 0 | CEJ was within the first marking |
| 1 | CEJ reached the second marking |
| 2 | CEJ reached the third marking |
| 3 | CEJ reached the fourth marking |
| 4 | CEJ reached beyond all markings |

CEJ: Cemento-enamel junction
