# Supplementary material for: Examining relationship between occupational acid exposure and oral health in workplace
Source: BMC Public Health. 2020 Sep 7;20:1371. doi: 10.1186/s12889-020-09496-6 (PMC7487460; doi:10.1186/s12889-020-09496-6)
Supplement: Supplementary file 2 — Additional file 2: Table S2. The oral hard/soft tissue indices for study survey [file 12889_2020_9496_MOESM2_ESM.doc]

**Supplemental table 2. The oral hard/soft tissue indices for study survey**

| Oral hard tissue | | | |
| --- | --- | --- | --- |
| Tooth erosion | | Dental caries | |
| No | Yes | No | Yes |
| Level 0 | Level 1 to 3 | DMFT=0 | DMFT>0 |
| Oral soft tissue | | | |
| Periodontal disease | | Loss of attachment | |
| No | Yes | No | Yes |
| CPITN=0 | CPITN>0 | LA=0 | LA>0 |
