# Supplementary material for: Examining relationship between occupational acid exposure and oral health in workplace
Source: BMC Public Health. 2020 Sep 7;20:1371. doi: 10.1186/s12889-020-09496-6 (PMC7487460; doi:10.1186/s12889-020-09496-6)
Supplement: Supplementary file 3 — Additional file 3. Questionnaire. English version of personnel health survey questionnaire [file 12889_2020_9496_MOESM3_ESM.doc]

Personnel Health survey questionnaire

**One: Basic information**

1. Height： cm；Weight： kg

2. Waistline: cm；Hip line： cm

3. Sex：Male □ Female □

4. Education：□ Elementary；□ High school；□ Vocational；□ College；□ More than college

**Second: Health behavior**

1. Did you have a smoking habit（cigarette use per day）？

□ Never smoked

□ Quit（almost half year）

□ Yes, Still smoking

2. Currently do have a daily drinking habit (Drink more than once a week at least)？

□ No

□ Quit（almost half year）

□ Yes, Still drinking

3. Having habit chewing betel nuts (Chewing betel nut than once a day at least)？

□ No

□ Quit（Quit eating betel nut for six months）

□ Yes, Still chewing

4. If you have a habit of hot springs？

□ No

□ Yes, Frequency: □1. Once a week □2. Once a month □3. Once for every months □4. Once in a year

**Third: Dietary habits and oral health**

Acidic diet：（Please recall the daily diet health, **select the following**）

| Food type | Intake frequency |
| --- | --- |
| Apple juice | □1. More than seven times a week □2. 4-7 times a week □3.1-3 times a week □4. Less than once a week. |
| Orange juice | □1. More than seven times a week □2. 4-7 times a week □3.1-3 times a week □4. Less than once a week. |
| Lemon juice | □1. More than seven times a week □2. 4-7 times a week □3.1-3 times a week □4. Less than once a week. |
| Grapefruit juice | □1. More than seven times a week □2. 4-7 times a week □3.1-3 times a week □4. Less than once a week. |
| Pineapple juice | □1. More than seven times a week □2. 4-7 times a week □3.1-3 times a week □4. Less than once a week. |
| Plum | □1. More than seven times a week □2. 4-7 times a week □3.1-3 times a week □4. Less than once a week. |
| Strawberry | □1. More than seven times a week □2. 4-7 times a week □3.1-3 times a week □4. Less than once a week. |
| Grape | □1. More than seven times a week □2. 4-7 times a week □3.1-3 times a week □4. Less than once a week. |
| Coffee | □1. More than seven times a week □2. 4-7 times a week □3.1-3 times a week □4. Less than once a week. |
| Black tea | □1. More than seven times a week □2. 4-7 times a week □3.1-3 times a week □4. Less than once a week. |
| Soda drinks (like Cola, soda, Sprite) | □1. More than seven times a week □2. 4-7 times a week □3.1-3 times a week □4. Less than once a week. |
| Wine (Red Wine、white wine) | □1. More than seven times a week □2. 4-7 times a week □3.1-3 times a week □4. Less than once a week. |

| Food type | Intake frequency |
| --- | --- |
| Cream cheese, fruit jelly | □1. More than seven times a week □2. 4-7 times a week □3.1-3 times a week □4. Less than once a week. |
| Noodles | □1. More than seven times a week □2. 4-7 times a week □3.1-3 times a week □4. Less than once a week. |
| Popcorn | □1. More than seven times a week □2. 4-7 times a week □3.1-3 times a week □4. Less than once a week. |
| Chocolate | □1. More than seven times a week □2. 4-7 times a week □3.1-3 times a week □4. Less than once a week. |
| Peanut | □1. More than seven times a week □2. 4-7 times a week □3.1-3 times a week □4. Less than once a week. |
| Beef, pork, fish meat | □1. More than seven times a week □2. 4-7 times a week □3.1-3 times a week □4. Less than once a week. |
| Sugar or Sugar substitutes | □1. More than seven times a week □2. 4-7 times a week □3.1-3 times a week □4. Less than once a week. |
| Nut | □1. More than seven times a week □2. 4-7 times a week □3.1-3 times a week □4. Less than once a week. |
| Distilled or pure water | □1. More than seven times a week □2. 4-7 times a week □3.1-3 times a week □4. Less than once a week. |
| Dried fish | □1. More than seven times a week □2. 4-7 times a week □3.1-3 times a week □4. Less than once a week. |
| Squid | □1. More than seven times a week □2. 4-7 times a week □3.1-3 times a week □4. Less than once a week. |
| Cheesecake | □1. More than seven times a week □2. 4-7 times a week □3.1-3 times a week □4. Less than once a week. |
| Yolk | □1. More than seven times a week □2. 4-7 times a week □3.1-3 times a week □4. Less than once a week. |
| Wheat grains | □1. More than seven times a week □2. 4-7 times a week □3.1-3 times a week □4. Less than once a week. |
| Milk, egg, tofu | □1. More than seven times a week □2. 4-7 times a week □3.1-3 times a week □4. Less than once a week. |
| Fried foods | □1. More than seven times a week □2. 4-7 times a week □3.1-3 times a week □4. Less than once a week. |

**Fourth: Condition of oral health**

1. Did you have gums bleeding within a year？ □0.No □1. Yes
2. Did you have any experience with toothache? □0. No □1. Yes
3. Did you have any teeth shake now？ □0.No □1. Yes
4. Did you open your mouth to breath？ □0.No □1. Yes
5. Did you often feel that you have bad breath？ □0.No □1. Yes
6. Did you feel sore teeth when you eat cold, hot, sour, sweet food or blowing cold wind □0. No □1.Yes
7. Did you limit the type of food you eat due to dental conditions, chewing or swallowing problems？(For example: unable to eat something due to dental caries)

□1.Never □2. Sometimes □3.Often

1. Overall, what do you think of the current dental health?

□1.Well □2.Fine 3.ordinary □4.Not good □5.Very bad

1. When did your last visit to go to the dental clinic?

□1.Within 6 months □2. Within a year □3. More than a year □4. More than 2 years

1. What was the most important reason for your last visit to the dentist (choose only one)？

□1.Relieve pain□2.Extraction □3. Fill the cavities □4.Dentures □5. Asthetic □6. Maintenance (dental cleaning)、inspection

□7.Other(please specify)____________________________________________________

1. Will you go regularly dental cleaning？

□0.No □1. Yes

1. When was your last dental cleaning？

□1.Within 6 months □ 2. Within a year □ 3.Within 2 years □4.Within 3 years

1. How many times have you been treated by a dentist in the last 6 months？

□1.No □2. Once □3. Twice □4. More than 3 times

1. Did you have experience in dental implants？

□0.No □1. Yes, the number of implants is (please specify) _________PCS

1. Did you have experience in orthodontics?

□0.No □1. Yes, how long before the correction (please specify) year ago

1. Did you have experience in dentures？

□0.No □1. Yes, how many dentures did you have (please specify) PCS

1. How many times did you brush your teeth everyday？

□1.No □2. Once □3. Twice □4. More than 2 times

1. When do you usually brush your teeth or clean your mouth？

□1.No brushing □2. After getting up in the morning and before going to bed at night □3. After getting up in the morning, before going to bed at night, and after meals (after each snack) □4. Before going to bed (after the last meal of the day)

1. In addition to brushing your teeth with a tooth-brush, what other ways do you use to clean your mouth？(Multiple choice)

□1.Inter dental brush □2. Dental floss stick □3. Floss with both hands □4. Chewing sugar free gum □5. Xylitol chewing gum □6. Mouthwash □7. Toothpick □8. Other □9. No

1. Do you have the habit of often using dental floss (non-floss stick) with both hands？

□0.No □1. Sometimes □2. Occasionally □3. Often □4. Almost after every meal

1. Do you have a toothbrush that uses soft bristles？

□0.No □1. Yes

1. What is your main way of brushing?

□1. Brush up and down □2. Brush left and right lateral □3. All have

**Fifth: Job background**

Current work history

1.When did you enroll the company? ______Year(s)_______Month(s)

2. Please fill in the units and work content that you have served during the company。

|  | Date | Work units | Work content | Chromium exposed |
| --- | --- | --- | --- | --- |
| 1 |  |  |  | □yes；□no |
| 2 |  |  |  | □yes；□no |
| 3 |  |  |  | □yes；□no |
| 4 |  |  |  | □yes；□no |
| 5 |  |  |  | □yes；□no |

**Sixth: Use of personal protective equipment (PPE)**

1. Please fill in the use of the following protective equipment (multiple choice)

| Type of protective equipment | Frequency of usage (Please choice the options as 2#) | Frequency of replacement (Please choice the options as 3#) |
| --- | --- | --- |
| - 1.Cotton masks | 1 2 3 4 5 6___________ | 1 2 3 4 5___________ |
| - 2.Paper masks | 1 2 3 4 5 6___________ | 1 2 3 4 5___________ |
| - 3.Carbon masks | 1 2 3 4 5 6___________ | 1 2 3 4 5___________ |
| - 4.N95 face mask | 1 2 3 4 5 6___________ | 1 2 3 4 5___________ |
| - 5.Respiratory mask | 1 2 3 4 5 6___________ | 1 2 3 4 5___________ |
| - 6.Powered air purifiers respirator (PAPR) | 1 2 3 4 5 6___________ | 1 2 3 4 5___________ |
| - 7.Cotton gloves | 1 2 3 4 5 6___________ | 1 2 3 4 5___________ |
| - 8.Plastic gloves | 1 2 3 4 5 6___________ | 1 2 3 4 5___________ |
| - 9.Apron | 1 2 3 4 5 6___________ | 1 2 3 4 5___________ |
| - 10.Safety goggles | 1 2 3 4 5 6___________ | 1 2 3 4 5___________ |
| - 11.other____________ | 1 2 3 4 5 6___________ | 1 2 3 4 5___________ |

#2.How often do you wear protective gear during work?

1.No need(0%) 2.Irregularly (25%) 3.sometimes (50%) 4.Regularly(75%) 5.Always(100%) 6.other(Please correspondent)

#3.How often do you change protective equipment?

1. More than two weeks does not change or only once 2. About a week (5-7 days) for once 3. Changed every 2-3 days 4.Changed every day 5. other(Please correspondent)

2.Whether to wear more than two kinds of respiratory protective equipment at work?

□ 1.no □ 2.yes
